# First Episode Psychosis and intensity of care after discharge : difference at two years between lost and maintained follow-up patients

**DOI:** 10.1192/j.eurpsy.2022.1964

**Published:** 2022-09-01

**Authors:** R. Bordas, E. Laffont, C. Jourdan, M. Pujol, L. Lamary

**Affiliations:** Ariège Couserans Hospital, Psychiatry, FOIX, France

**Keywords:** schizophrénia, First Episode, Psychosis, Disengagement

## Abstract

**Introduction:**

Disengagement from care in early psychosis is frequent. In outpatient general psychiatric services rates range from 17 % to 60 %. In early intervention programs, rates range from 14 % to 33 % at two years. In Europe, a study reported an initial drop out rate at 48 %.

**Objectives:**

Measure intensity of care during two years after first hospitalization in a schizophrenia spectrum disorder population. Search for a difference between lost and maintained follow-up patients.

**Methods:**

A monocentric retrospective study was conducted. All patients aged 16 to 30 with at least one hospitalization for schizophrenia spectrum disorder from January 2013 to December 2018 in CHAC were included. First hospitalization medical charts and all (social, nurse, psychologist, psychiatrist) outpatient appointments were assessed. A monthly mean of all appointments (MMA) was calculated for each patient. Lost or maintained groups at two years were compared with a Mann-Whitney test.

**Results:**

Among 48 patients, 52,1 % (N=25) disengaged from initial follow up within 2 years. The MMA for (N=46) patients was 1,45 (SD 1,35), 0,5 (SD 0,33) for psychiatrists. For lost patients, the MMA was 1,35 (SD 1,40) compared to 1,55 (SD 1,32) for maintained. No significant difference was found : U=229,50 p=0,45.

**Conclusions:**

At two years, care appears more intensive for maintained patients than for lost ones, but no significant difference was found.

**Disclosure:**

No significant relationships.

